# Brain Health and Dementia Care Services: Community-Led Innovation With and for Indigenous Peoples

**DOI:** 10.1093/geroni/igaf122.1927

**Published:** 2025-12-31

**Authors:** Mary Wyman, Sarah Punshon, Jordan Lewis

## Abstract

Compared to other groups of older adults, Indigenous peoples are at higher risk for developing cognitive disorder as they age, due to health and healthcare inequities. However, indigenous communities also have a rich history of resilience and creativity, as well as many cultural traditions that support brain health and best practice care for persons with dementia. This symposium brings together several indigenous-focused projects across the US working within collaborative community-engaged frameworks, all of which center meaningful interactions with community members throughout the scholarly process to attain goals of improved community understanding of brain health and dementia, cultural tailoring of best practice models, and innovative approaches to improve services to help Indigenous elders age in place. Thompson et al. report on a community-led effort to enhance the GSA-developed Kickstart, Assess, Evaluate, and Refer (KAER) model for brain health education, tailoring it for elders in the Meskwaki Settlement in Tama County, Iowa. Kim and colleagues joined with community members to develop accessible, community-driven online educational resources focused on dementia prevention and supporting dementia caregivers in remote Alaska Native communities. Punshon and co-authors describe a novel partnership between multiple healthcare systems to support implementation of specialty dementia diagnosis and management services via telehealth within a rural Native American community. All presentations will outline innovative, successful strategies to strengthen community-held knowledge and practices that can be emphasized and built upon to expand resources for dementia prevention and care.

Indigenous Peoples Interest Group Sponsored Symposium

